# A Comparative Study of Molecular Structure, p*K*a, Lipophilicity, Solubility, Absorption and Polar Surface Area of Some Antiplatelet Drugs

**DOI:** 10.3390/ijms17030388

**Published:** 2016-03-19

**Authors:** Milan Remko, Anna Remková, Ria Broer

**Affiliations:** 1 Department of Pharmaceutical Chemistry, Faculty of Pharmacy, Comenius University in Bratislava, Odbojarov 10, SK-832 32 Bratislava, Slovakia; 2 Department of Internal Medicine, Faculty of Medicine, Slovak Medical University, Limbová 12, SK–833 03 Bratislava, Slovakia; aremkova@szu.sk; 3 Department of Theoretical Chemistry, Zernike Institute for Advanced Materials, University of Groningen, Nijenborgh 4, 9747 AG Groningen, The Netherlands; R.Broer@rug.nl

**Keywords:** antiplatelet agents, molecular structure, solvent effect, p*K*a, lipophilicity, solubility, absorption, polar surface area

## Abstract

Theoretical chemistry methods have been used to study the molecular properties of antiplatelet agents (ticlopidine, clopidogrel, prasugrel, elinogrel, ticagrelor and cangrelor) and several thiol-containing active metabolites. The geometries and energies of most stable conformers of these drugs have been computed at the Becke3LYP/6-311++G(d,p) level of density functional theory. Computed dissociation constants show that the active metabolites of prodrugs (ticlopidine, clopidogrel and prasugrel) and drugs elinogrel and cangrelor are completely ionized at pH 7.4. Both ticagrelor and its active metabolite are present at pH = 7.4 in neutral undissociated form. The thienopyridine prodrugs ticlopidine, clopidogrel and prasugrel are lipophilic and insoluble in water. Their lipophilicity is very high (about 2.5–3.5 log*P* values). The polar surface area, with regard to the structurally-heterogeneous character of these antiplatelet drugs, is from very large interval of values of 3–255 Å^2^. Thienopyridine prodrugs, like ticlopidine, clopidogrel and prasugrel, with the lowest polar surface area (PSA) values, exhibit the largest absorption. A high value of polar surface area (PSA) of cangrelor (255 Å^2^) results in substantial worsening of the absorption in comparison with thienopyridine drugs.

## 1. Introduction

Arterial thrombosis is a common cause of ischemic stroke, myocardial infarction and limb gangrene. This disease is a leading cause of morbidity and mortality in developed countries [1]. Atherothrombosis is essentially a platelet-driven process [2,3,4]. Pharmaceutical research has focused on the development of antiplatelet drugs targeting key pathways of platelet activation [2,3,4,5,6]. Three classes of antiplatelet agents are currently approved for clinical use: (i) cyclooxygenase-1 (COX-1) inhibitors (aspirin); (ii) adenosine diphosphate (ADP) P2Y12 receptor antagonists (clopidogrel); and (iii) glycoprotein IIb/IIIa inhibitors (abciximab) [2,3]. Besides the cornerstone of antiplatelet therapy, aspirin, also thienopyridines (ticlopidine, clopidogrel, prasugrel), especially in dual therapy with aspirin, have become standard antiplatelet therapies [7]. Owing to known clinical limitations of traditional drugs, like clopidogrel, a number of novel drugs are currently at various stages of clinical development. Three agents, prasugrel, cangrelor and ticagrelor, were already approved for antiplatelet therapy.

Ample experimental biological evidence is available for the relationship between the chemical and biological properties of new antiplatelets targeting the P2Y12 platelet receptor and their biological activity. Nevertheless, experimental studies engaged in the systematic comparison of the physicochemical and pharmacokinetic parameters of these antiplatelet agents are not very well characterized. The absence of experimental structural data of new synthetic antiplatelets targeting the P2Y12 platelet receptor presents a challenge to the application of molecular modeling methods with the aim to enhance our understanding of the subtle pharmacological effects of these antithrombotics.

**Figure 1:**
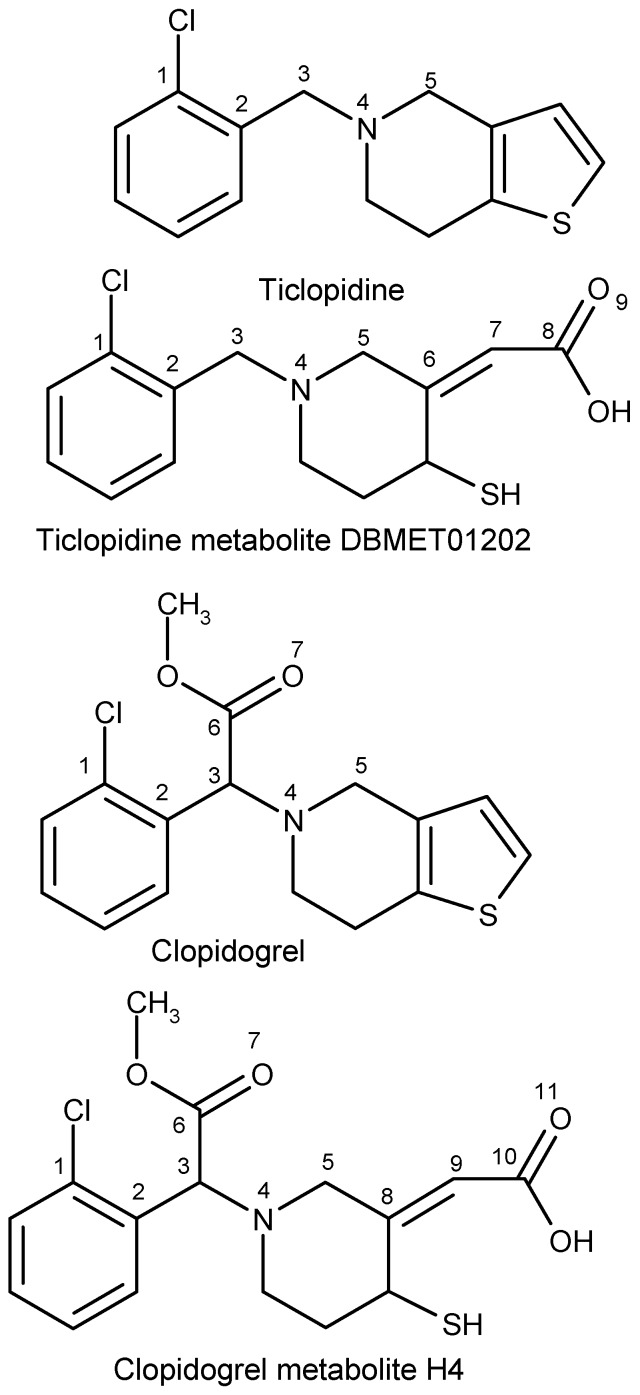

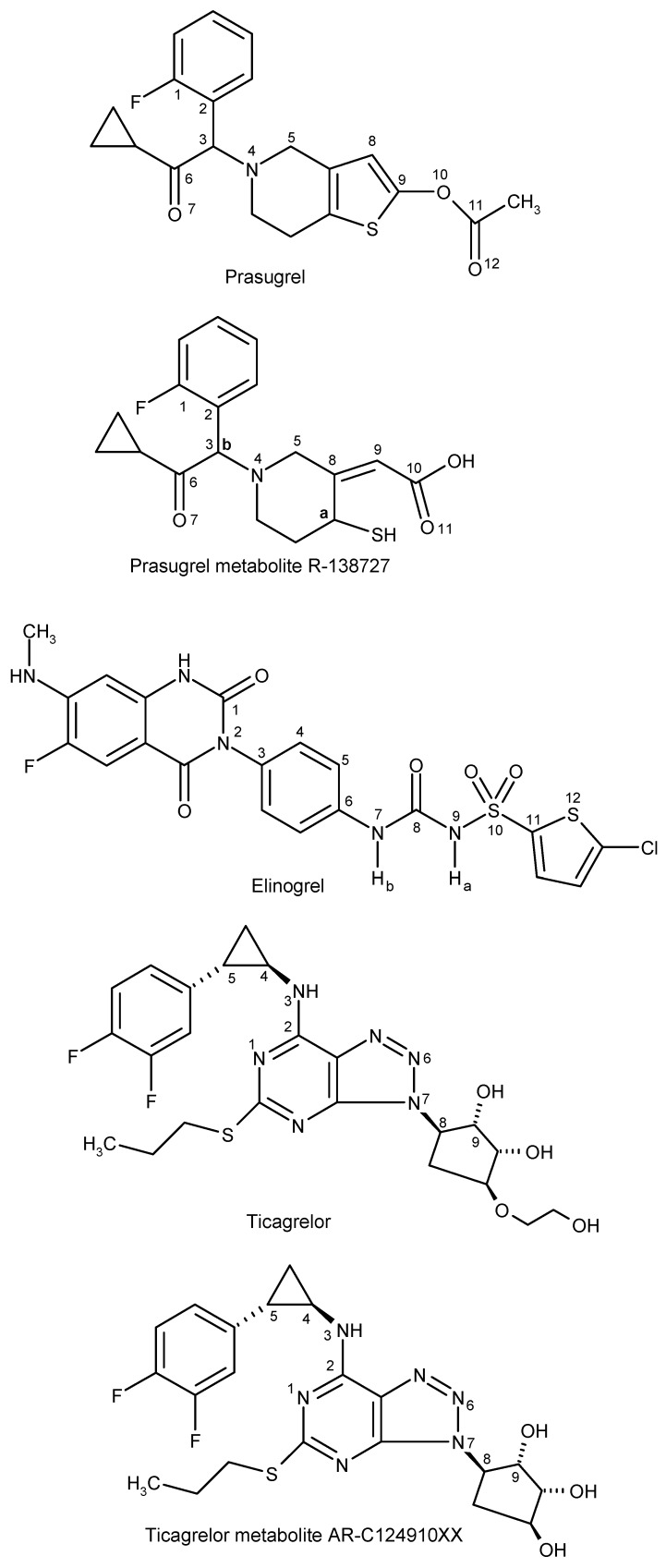

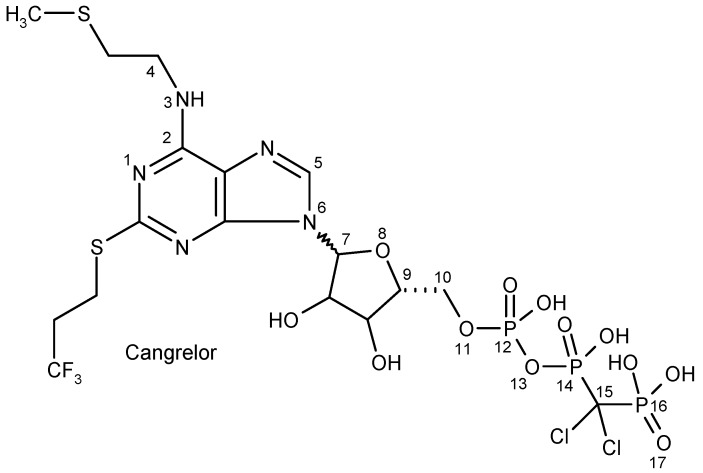
Structure and atom labeling in the antiplatelet drugs investigated.

Here, we use density functional methods [8,9,10] to investigate the structure of six P2Y12 platelet receptor inhibitors (approved drugs ticlopidine, clopidogrel, prasugrel, cangrelor and ticagrelor) and experimental drug elinogrel, respectively (Figure 1). The polarizable continuum method was used to account for the effect of solvent on the equilibrium conformation of these drugs. Our interest is also focused on the evaluation of the important physicochemical properties of drugs studied (p*K*a, lipophilicity, solubility, absorption and polar surface area). The results of the molecular modeling investigations of these antiplatelets were compared to the available experimental structural data and discussed in relation to the present theories of action of these agents.

## 2. Results and Discussion

### 2.1. Molecular Structures

The prediction of the equilibrium structure of drugs is one of the main goals of modern quantum chemistry [11]. Quantum chemical calculations are now an effective tool in drug design and development. They are used for an accurate determination of molecular structures and properties for use in a wide variety of computer-aided drug design (CADD) studies [12,13].

The relative orientations of important functional groups of the antiplatelet agents studied here are defined by dihedral angles (α, β, γ, δ, ε, ζ, η, ν, θ, μ and ξ; Table 1). The overall view of the optimized geometry of the drugs is presented in the Supplementary Material. The relevant values of the torsion angles are reported in Table 1. In all cases studied, except for cangrelor tetrasodium, the water had only a small effect on the equilibrium geometry of the antiplatelet agents investigated (Table 1). Table 2 shows the solvation energies obtained from combining calculations performed in a vacuum with calculations based on the solvation method used. B3LYP/6-311++G (d,p) calculations in water yielded conformers not significantly different from the isolated molecules, and hence, only the geometries of the isolated molecules are reported in the Supplementary Material. The studied antiplatelet drugs exhibited considerable stability in water, as expected (Table 2). The solvation energy (energy difference between the gas phase and solvated phase) is largest for cangrelor tetrasodium (−1316 kJ/mol), since it holds a considerable dipole moment and the ionic character of sodium bonds.

### 2.2. Ticlopidine

**Figure 2:**
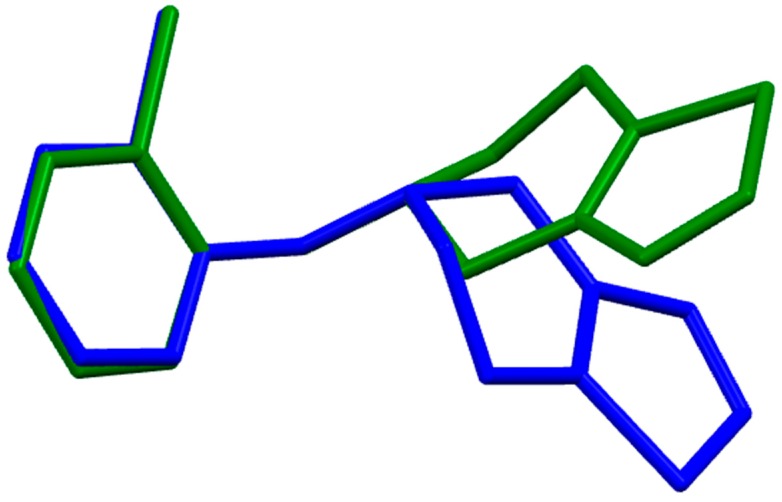
Molecular superimposition of the Becke3LYP optimized structure of ticlopidine (green) and ticlopidine from the co-crystal with cytochrome P450 2B4, PDB.2KW4 (blue). For simplicity, the hydrogen atoms are not shown.

Ticlopidine (5-[(2-chlorophenyl)methyl]-4,5,6,7-tetrahydrothieno[3,2-c]pyridine) is the first clinically useful thienopyridine derivative that inhibits platelet activation and aggregation by irreversibly blocking the ADP P2Y12 receptor [19]. Ticlopidine is a prodrug that in the body exhibits a complex metabolism [20]. The active metabolite (Figure 1) is a reactive molecule, which irreversibly binds to its receptor. The pharmacologically-active metabolite UR-4501 ([1-[(2-chlorophenyl)methyl]-4-mercapto-3-piperidinylidene]acetic acid) possesses a free thiol group, which is involved in the disulfide bond with extracellular cysteine residues and inactivating the P2Y12 ADP receptor [19,20]. Both ticlopidine and its active metabolite are structurally rigid molecules with a 3D structure governed by the mutual position of the phenyl and thienopyridine rings (dihedral angles α[C(1)–C(2)–C(3)–N(4)] and β[C(2)–C(3)–N(4)–C(5)]; Table 1). These moieties exist in the gas phase and in water solution in a mutual *gauche* arrangement. Due to crystal packing forces and protonation of the nitrogen atom, a different conformation was observed for structurally-related ticlopidine hydrochloride with dihedral angles α[C(1)–C(2)–C(3)–N(4)] and β[C(2)–C(3)–N(4)–C(5)] equal to −98 and 66 degrees, respectively [15]. The values of these dihedral angles in the biological environments are quite different (Table 1). The coordination of the ticlopidine to the cytochrome P450 2B4 metabolizing enzyme [14] leads to a *trans* arrangement of the phenyl ring and the thienopyridine moieties (dihedral angle β[C(2)–C(3)–N(4)–C(5) = 179.5°); see Figure 2. A different situation exists with the biologically-active metabolite of ticlopidine UR-4501 [20]. The optimized geometry corresponds to the structure in which the planar benzyl group and piperidine moieties are in a mutual *gauche* position (dihedral angle β[C(2)–C(3)–N(4)–C(5) is about −72°; Table 1). The piperidine moiety prefers a chair conformation with the *equatorially*-oriented N–C bond and C=C–CO_2_H substituent. The thiol group is in *axial* position (Figure S1, Supplementary Material). The configuration of the unsaturated carboxylic acid moiety –CH=CH–COOH (dihedral angle δ[C(6)–C(7)–C(8)–O(9)]) may be in *E* and/or *Z* orientation. Both isomers of this metabolite were examined. The *Z* isomer (δ[C(6)–C(7)–C(8)–O(9)] = −8.1°) was found to be by 14.9 (gas-phase) and 9.2 kJ/mol (aqueous phase) more stable. The *Z* conformation of the C=O group with respect to the double bond of the conjugated side chain was also found experimentally for structurally-related acrylic acid [21].

#### 2.2.1. Clopidogrel

**Figure 3:**
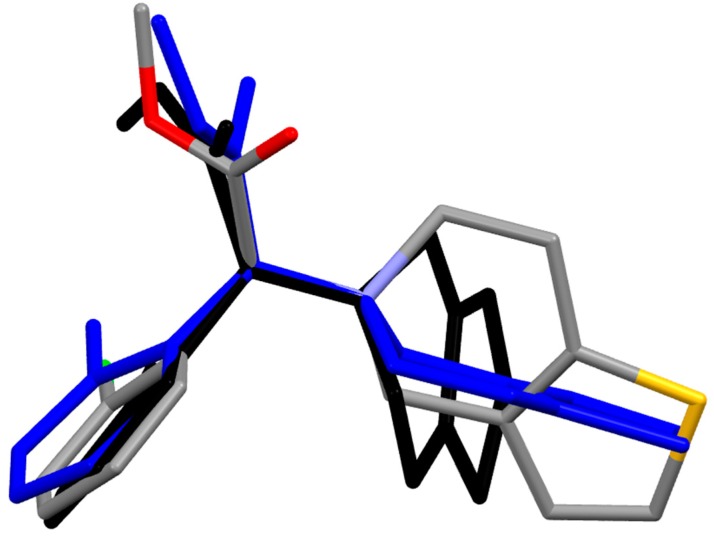
Molecular superimposition of the Becke3LYP-optimized molecular structure of clopidogrel (color by connected atoms), clopidogrel from the cocrystal with the cytochrome P450 2B4 active site PDB.3ME (blue) and clopidogrel from the cocrystal with the cytochrome P450 2B4 active site mutant F297A PDB.4H1N (color by connected atoms, carbon gray, oxygen red, sulfur yellow). For simplicity, the hydrogen atoms are not shown.

Clopidogrel ((*S*)-methyl α-(4,5,6,7-tetrahydrothieno[3,2-c]pyridin-5-yl)-α-(o-chlorophenyl)acetate) is a thienopyridine molecule that structurally resembles ticlopidine (Figure 1). Like ticlopidine, its (*S*)-isomer has antiplatelet and antithrombotic effects [19]. Clopidogrel itself is a prodrug, which needs hepatic metabolization to be active. Its active metabolite (2*Z*)-{1-[(1*S*)-(2-chlorophenyl)-2-methoxy-2-oxoethyl]-4-sulfanyl-3-piperidinylidene}acetic acid is permanently bound to its receptor and is associated with a long duration of action [22,23,24]. The biologically-active metabolite is a highly reactive species. Due to the highly unstable character, it was impossible to isolate it from human microsomal incubations in sufficient amounts to determine its molecular structure by X-ray crystallography [24]. In comparison with ticlopidine, clopidogrel has a different substituent pattern at the benzylic carbon. Substitution of one hydrogen of this carbon by the methyl ester group resulted in a new drug, with increased potency and reduced side effects as compared to its parent ticlopidine [19,23]. The 3D structural motif of clopidogrel containing one chiral center is characterized by the existence of two enantiomers. The (*S*)-enantiomer is, according to our calculations, by 10.2 (gas-phase) and 9.6 kJ/mol (aqueous environment) the most stable species. The mutual conformation of clopidogrel around the C(2)–C(3) and C(3)–N(4) single bonds connecting the phenyl and thienopyridine moieties is almost the same as in the parent ticlopidine (Table 1). The methyl ester group is, with respect to the plane defined by C(2)–C(3)–N(4) atoms, rotated by about 88° (dihedral angle δ[C(2)–C(3)–C(6)–O(7)]) and oriented away from the bulky scaffold of the molecule (Figure S1, Supplementary Material). The solid-state structure was experimentally determined for the salt of clopidogrel with sulfuric acid only [17,25]. Clopidogrel hydrogen sulfate exhibits polymorphism: different polymorphs exist in different conformations [17]. The computed gas-phase conformation of clopidogrel is close to the monoclinic form of clopidogrel hydrogen sulfate obtained from powder diffraction data [17]; see Table 1. The solvent effect practically does not change the spatial structure of the isolated molecule of clopidogrel. CYP450 enzymes are involved in both oxidative reactions of clopidogrel’s biotransformation to its active metabolite. The binding properties of clopidogrel towards its metabolizing enzymes were investigated using cytochrome P450 2B4 [14] and P450 2B4 active site mutant F297A [16] as targets. Clopidogrel exists in both complexes in divergent conformations, which differ from those calculated for the isolated molecule (Table 1) with the thienopyridine moiety differently distributed in the space for bound and unbound clopidogrel (Figure 3).

The gas-phase conformation of the phenyl and piperidine groups of the clopidogrel metabolite defined by the rotation around the C(2)–C(3) and C(3)–N(4) single bonds is close to the conformation found in parent clopidogrel (Table 1). The conformation of the piperidine moiety copies the stable conformation of the same group of ticlopidine metabolite. The configuration of the –CH=CH–COOH group (dihedral angle δ[C(6)–C(7)–C(8)–O(9)]) is, like for the ticlopidine moiety, Z. The *E* isomer is by 15.4 (gas-phase) and 9.4 kJ/mol (aqueous environment) less stable. The (*S)* configuration at the benzylic carbon and the *Z* configuration at the C=C double bond of the active metabolite were also confirmed experimentally [24].

#### 2.2.2. Prasugrel

Prasugrel, a prodrug, (5-[2-cyclopropyl-1-(2-fluorophenyl)-2-oxoethyl]-4,5,6,7-tetrahydrothieno[3,2-c]pyridin-2-yl acetate) is a novel and potent irreversible thienopyridine inhibitor of platelet aggregation [26,27]. Its conformational structure is, like in ticlopidine and clopidogrel, determined by dihedral angles α, β, δ, ε and ζ (Table 1, Figure 1). The benzylic carbon atom is substituted with a cyclopropyl carbonyl moiety. The thiophene ring of prasugrel contains an acetoxy group, and the phenyl ring caries a fluoro substituent (Figure 1). These changes in composition, in comparison with its predecessor clopidogrel, result in a stronger inhibitor of ADP-induced platelet aggregation and in more consistent and more rapid action [28,29]. Prasugrel’s (*R*) and (*S*) enantiomers undergo spontaneous racemization in solution, and it is administered as a racemate in the form of hydrochloride salt. The DFT calculations show that (*S*)-prasugrel is by 10.1 and 7.8 kJ/mol (gas-phase and aqueous solution) a more stable enantiomer. The conformational structure of (*S*)-prasugrel mimics the structure of the thienopyridine scaffold (Table 1). The equilibrium conformation of the acetoxy group is stabilized by means of intramolecular non-bonded interaction between oxygen and sulfur atoms (Figure 2). In this molecule, the Becke3LYP/6–311++G(d,p) calculated length (2.78 Å) between non-bonded S···O atoms of the acetoxy group and the thiol ring is shorter than the sum of the van der Waals radii for oxygen and sulfur atoms (3.32 Å) [30]. This intramolecular non-bonded S···O interaction has been observed in a large number of organosulfur compounds [31,32,33]. The aqueous environment does not have an important effect on the overall shape of prasugrel (Table 1). The gas-phase conformation of prasugrel and the solid state structure determined by X-ray crystallography [18] are close to each other, indicating that the crystal packing forces do not play any important role (Table 1, Figure S1 in the Supplementary Material).

As a prodrug, prasugrel is metabolized into several metabolites [28,29]. Just one of them, R-138727 ((2Z)-{1-[2-cyclopropyl-1-(2-fluorophenyl)-2-oxoethyl]-4-sulfanyl-3-piperidinylidene}acetic acid) is a biologically-active drug, which irreversibly blocks the P2Y12 ADP receptor. Our calculations show that the active metabolite has a Z configuration at the C(8)–C(9) double bound. This isomer is by 15.2 (gas phase) and 9.4 kJ/mol (solvated phase) more stable (dihedral angle ε[C(5)–C(8)–C(9)–C(10)]; Table 1) than the *E* isomer. Experiments showed that the metabolism of prasugrel in humans is stereoselective, and four stereoisomers of R-138727 were separated by analytical techniques [34]. R-138727 contains two chiral atoms and may exist in four stereoisomeric forms, (*R*,*R*), (*S*,*S*), (*R*,*S*) and (*S*,*R*), respectively. We performed full geometry optimizations of these stereoisomers and calculated their relative energy stability in both the gas phase and the aqueous state (Table 3). The (*S*,*S*) stereoisomer is the most stable species followed by the (*S*,*R*) one. In these isomers, the –SH group is in the *axial* position. Two substantially less stable (*R*,*R*) and (*R*,*S*) enantiomers, identified in the human plasma samples as prevailing species, cannot exist in both environments with any probability. The calculated populations at 310.2 K for the (*S*,*S*), (*S*,*R*), (*R*,*S*) and (*R*,*R*) stereoisomers are in the ratio of 99:1:0:0 (in both gas-phase and solvated phase). However, the drug metabolism pathways for chiral drugs are different for the individual enantiomers [35]. The same is true for the enzymatic formation of R-138727, which is a stereo-selective process [34], and most pharmacologically-active antiplatelet isomers represent (*R,R*) and (*R,S*) enantiomers [34].

#### 2.2.3. Elinogrel

**Scheme 1:**
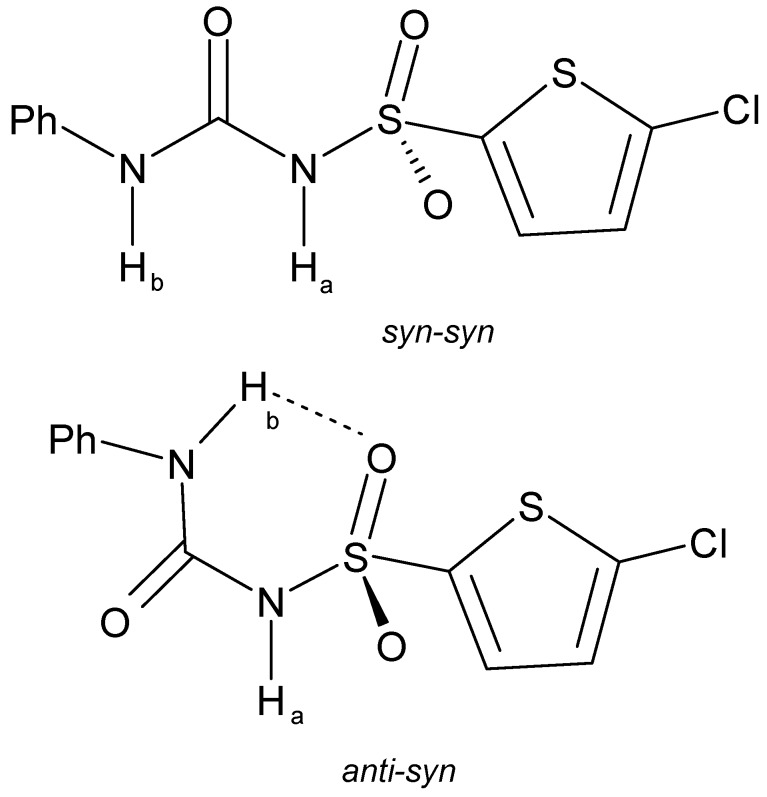
Stereoisomers of elinogrel.

Elinogrel (*N*-[4-(6-fluoro-7-methylamino-2,4-dioxo-1,4-dihydro-2*H*-quinazolin-3-yl)phenyl]-*N*′-[(5-chlorothiophen-2-yl)sulfonyl]urea) is a sulfonylurea derivative, which entered into clinical trials acting as a reversible ADP P2Y12 inhibitor [36]. Its conformational structure is governed by the stereochemistry of the sulfonylurea moiety. As far as *N*,*N*-disubstituted ureas are concerned, the urea skeleton in the solid state is planar, and the urea moiety has a *syn-syn* conformation [37]. A different situation exists with sulfonylurea derivatives. Based on the extensive conformational studies of sulfonylurea drugs, two stable conformations were identified, *syn-syn* and *anti-syn* (Scheme 1) [38,39]. The *anti-syn* conformer is in the gas-phase and aqueous solution by 23 and 7.7 kJ/mol more stable. The *anti-syn* conformer is characterized by an intramolecular hydrogen bond N–H···O=S with an H···O bond length of about 2 Å (Figure S1, Supplementary Material). This intramolecular H-bond is sufficient to overcome the unfavorable *cis* amide conformation –C(8)–N(9)H_a_ in this conformer. The N–H···O=S interaction has been displayed also for solid state structurally-related gliquidone [40]. The planar quinazolinedione and phenyl moieties exist in the stable perpendicular arrangement (the dihedral angle α[C(1)–N(2)–C(3)–C(4)] is about 90°). The known planarity of the urea group –NH–(C=O)–NH– in solid state was also confirmed in the gas-phase and aqueous solution (Table 1). However, the observed non-planarity of phenylurea moiety observed in crystals of substituted phenylureas [37,41] was not found in our DFT calculations. The molecular structure of this fragment of elinogrel exhibits a planar conformation with an electronically-delocalized character of the phenylurea scaffold (dihedral angle β[C(5)–C(6)–N(7)–C(8)]; Table 1). The thiophene and sulfonylurea groups are perpendicularly oriented to each other, and the molecule exhibits an L-shaped conformation (Figure S1, Supplementary Material).

#### 2.2.4. Ticagrelor

Ticagrelor ((1*S*,2*S*,3*R*,5*S*)-3-[7-[[(1*R*,2*S*)-2-(3,4-difluorophenyl)cyclopropyl]amino]-5-(propylthio)-3*H*-1,2,3-triazolo[4,5-d]pyrimidin-3-yl]-5-(2-hydroxyethoxy)-1,2-cyclopentanediol) is an orally-active P2Y12 antagonist [42] approved by the FDA in 2011. Structurally, it is an analog of ATP that reversibly binds to the P2Y12 platelet receptor. The triazolopyrimidine scaffold and thiopropyl substituent are almost coplanar. The cyclopropyl-difluorophenyl moiety is in *anticlinal* position to the central triazolopyrimidine core (the dihedral angle β[C(2)–N(3)–C(4)–C(5)] is about −151°). Cyclopentanediol and triazolopyrimidine are in a mutual *anticlinal* position (the dihedral angle γ[N(6)–N(7)–C(8)–C(9)] is about 120–130°; Table 1). An aqueous solution does not have any appreciable effect on the stable conformation calculated. A very similar conformation was also found for the structurally-related active metabolite of ticagrelor (AR-C124910XX) formed by *O*-deethylation [43] (Table 1). All calculations were carried out with the chiral centers’ conformation corresponding to the biologically-active stereoisomers of ticagrelor and its metabolite (Figure S1, Supplementary Material).

#### 2.2.5. Cangrelor

**Figure 4:**
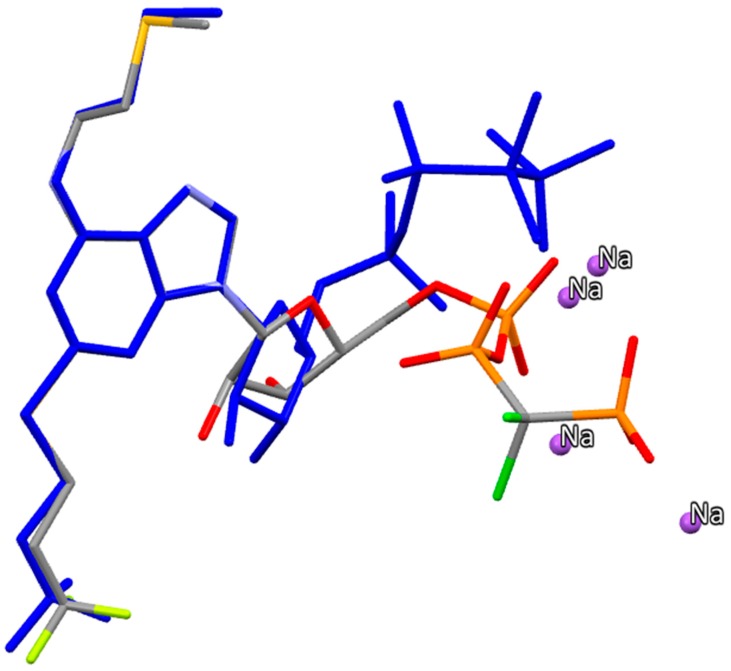
Molecular superimposition of the Becke3LYP optimized molecular structure of cangrelor (blue) and its sodium salt cangrelor tetrasodium (color by connected atoms, carbon gray, oxygen red, fluorine and chlorine green, sulfur yellow, phosphorus orange). For simplicity, the hydrogen atoms are not shown.

Cangrelor ([dichloro({[({[(2*R*,3*S*,4*R*,5*R*)-3,4-dihydroxy-5-(6-{[2-(methylsulfanyl)ethyl]amino}-2-[(3,3,3-trifluoropropyl)sulfanyl]-9*H*-purin-9-yl)oxolan-2-yl]methoxy}(hydroxy)phosphoryl)oxy](hydroxy)phosphoryl})methyl]phosphonic acid) is the first ATP analogue containing a purine core in clinical praxis, approved in 2015. It is a reversible competitive antagonist of P2Y12 receptors [42,44]. The rigid cangrelor core purine is at the C-2, C-6 and N-9 positions substituted by a flexible substituent. The conformation of the biologically-active stereoisomer is defined by 16 rotatable bonds. The important dihedral angles (α, β, γ, δ, ε, ζ, η, ν, θ, μ and ξ) are presented in Table 1. Both aliphatic substituents of the pyrimidine part of the molecule were considered in the stable extended conformations and situated in the plane of the purine scaffold. The purine and ribose moieties are in mutual *gauche* conformation (dihedral angle β[C(5)–N(6)–C(7)–O(8)]; Table 1) stabilized via the intramolecular hydrogen bond between the adjacent C–OH group of ribose and the N-3 nitrogen atom of the purine moiety with a OH···N distance of 1.98 Å (Figure S1, Supplementary Material). Such an intramolecular hydrogen bond was also observed experimentally in the adenosine hydrochloride [45]. The phosphate end group is in the folded conformation stabilized by OH···O=P hydrogen bonds. Cangrelor is in clinical praxis used in the form of its tetrasodium salt (tetrasodium 5′-*O*-[({[dichloro(phosphonato)methyl]phosphinato}oxy)phosphinato]-*N*-[2-(methylsulfanyl)ethyl]-2-[(3,3,3-trifluoropropyl)sulfanyl]adenosine). The molecular structure of sodiated cangrelor is completely different (Figure 4). The purine and ribose rings are in mutual quasiplanar conformation (the dihedral angle β[C(5)–N(6)–C(7)–O(8)] is about 18°). Salt formation of acidic phosphate groups leads to the dramatic change of the equilibrium conformation of the phosphate moiety of cangrelor (dihedral angles α, β, γ, δ, ε, ζ, η, ν, θ, μ and ξ; Table 1). The coordination of the anionic phosphate groups with sodium cations results in conformational stabilization of this part of cangrelor by means of bidentate and/or tridentate coordination (Figure S1, Supplementary Material). Aqueous solution has an important effect on the equilibrium conformation of the triphosphate group of tetrasodium salt, reflected in large changes of about 10–20° of torsional angles ζ, η, ν, θ and ξ; see Table 1.

### 2.3. Acidity and Basicity

The antiplatelet agents investigated carry an acidic and/or basic center, and at the physiological pH, they may exist as neutral drugs and/or ionized forms. Because dissociation and/or ionization plays an important part in the partition of drugs and their interaction with a receptor, it is important to know their acido-basic characteristics. In the absence of published experimental p*K*a values, the calculated data represent the basic characteristics about their acido-basic properties in water solution. The calculated macro p*K*as are shown in Table 4. Of the indirect irreversible thienopyridine drugs, ticlopidine is the most basic molecule. The basicity decreases after its metabolization in the liver. Therapeutically-active metabolites of ticlopidine, clopidogrel and prasugrel are organic acids with p*K*as in the range of 3–3.4, and at pH = 7.4, they exist in the dissociated form only. The acidic sulfonamide group of the direct antiplatelet agent elinogrel is at physiological pH completely dissociated, and its acidity (p*K*a = 4.3) is comparable with the acidity of other sulfonylurea drugs [38]. Both ticagrelor and its active metabolite are present at pH = 7.4 in the neutral undissociated form. Cangrelor contains three acidic phosphate groups with p*K*as in the range of 0.33–0.59, which are completely dissociated at blood pH (Table 4).

### 2.4. Lipophilicity, Solubility, Absorption, Polar Surface Area and “Rule of Five” Properties

The calculated lipophilicity (log*P*) and solubility (log*S*) parameters are shown in Table 5. The computational methods used (XLOGP2, AClog*S*) are available via the Virtual Computational Chemistry Laboratory website (http://www.vcclab.org). The methods chosen for the evaluation of lipophilicity, solubility and absorption were selected on the basis of the good performance of such methods for a larger number of antithrombotics belonging to different pharmacological groups [46,47]. The thienopyridine prodrugs ticlopidine, clopidogrel and prasugrel are lipophilic and insoluble in water. Their lipophilicity is very high (log*P* values of about 2.5–3.5), and in drug formulations, they exist in the form of water-soluble pharmaceutical salts. Prodrug activation in thienopyridines results in lowering of lipophilicity in comparison with parent prodrugs and in improving their solubility characteristics (Table 5). The active metabolites of the prodrugs (ticlopidine, clopidogrel and prasugrel) and drugs elinogrel and cangrelor are completely ionized at pH 7.4 (Table 5). For such species, as an index of lipophilicity, the distribution coefficient, log*D*, is used. The log*D* values were calculated from the computed log*P* (XLOGP2 method) and p*K*a (SPARC) using the equation log*D* = log*P* − log (1+10^p*H*−p*K*a^) and pH = 7.4 [48]. The calculated log*D*_7.4_ values for these ionizable species were very low (less than −0.9), indicating their high solubility, low permeability and metabolism. The intrinsic solubility in the neutral state (Log*S*) is a measure of a compound’s solubility (*S*). The parent prodrugs of antiplatelets studied are slightly soluble in water; nevertheless, their solubility is increased after metabolization activation (Table 5). The computed intrinsic solubility of three thienopyridine active metabolites, between 0.2 and 0.7 g/L, is enough for fast absorption. The orally-acting drugs ticlopidine, clopidogrel, prasugrel and ticagrelor appear to be rapidly absorbed [43,49]. The calculated percentages of absorption (%ABS), polar surface area (PSA) and Lipinski parameters of the investigated drugs are reported in Table 5. The percentage of absorption (%ABS) was calculated using the equation: %ABS = 109 − 0.345 PSA [50]. The fragment-based method of Ertl and coworkers was used for the calculation of the polar surface area (PSA) [51]. The calculated %ABS for orally-active drugs is very high and correlates well with the experimentally-observed good absorption of these drugs [43,49]. Cangrelor and elinogrel are direct and reversible P2Y12 platelet receptor inhibitors, which violate the “rule of five” (the molecular weight is too high). Cangrelor’s hydrogen bonding capacity over the limits based on Lipinski rules (27 hydrogen bonding acceptor and donor groups, respectively) is closely related to its low lipophilicity and highly polar character expressed by the large polar surface area of about 256 Å^2^ (Table 5). The calculated percentages of the absorption of intravenously-acting elinogrel and cangrelor are, on the other hand, lower. The polar surface areas, owing to the structurally-heterogeneous character of these antiplatelet drugs, exhibit very large intervals of values (Table 5). Thienopyridine prodrugs, like ticlopidine, clopidogrel and prasugrel, with the lowest PSA values, exhibit the largest absorption.

### 2.5. Selection Criteria for Antiplatelets Targeting the P2Y12 Platelet Receptor

The drug-like properties of prodrugs and drugs of antiplatelets studied are reported in Table 6. The data were collected for orally-acting drugs only. The first three molecular descriptors (*M*w, clog*P*, clog*S*) describe, together with the conformational studies presented in the Section 2.1 of this paper, important physicochemical descriptors of drug-like antiplatelets. The polar surface area is an important parameter regarding absorption. These molecular descriptors were originally designed to predict whether compounds will have absorption problems even before the design of prodrugs. Selected molecular descriptors for corresponding precursors are also reported in Table 6. In general, the range of molecular descriptors studied is wider for prodrugs. The thienopyridine prodrugs ticlopidine, clopidogrel and prasugrel have exhibited higher absorption levels than their metabolites. The percent of absorption of the precursors is, however, sufficiently high to enable a good absorption and a subsequent covalent bond formation with the P2Y12 platelet receptor. Ticagrelor and its active metabolite show identical absorption profiles with a similar hydrogen bonding capacity, structural flexibility and polar surface area. However, both ticagrelor and its biologically-active metabolite are allosteric antagonists that reversibly block the platelet P2Y12 purinergic receptor and represent a new chemical class called cyclopentyltriazolopyrimidines [42].

Selection criteria for drug-like properties of antiplatelets targeting the P2Y12 platelet receptor were designed and may be used for the selection of new compounds: inhibitors of the antiplatelet P2Y12 receptor. These criteria may be especially useful for the selection of new candidates with suitable physicochemical parameters. Lipinski’s “rule of five” originally applied for the design of drugs should be effectively applied also for prodrugs serving as one of the separation processes between prodrugs and drugs.

## 3. Computational Methods

Density functional theory (DFT) calculations of ticlopidine, clopidogrel, prasugrel, elinogrel, ticagrelor and cangrelor (Figure 1) were carried out by means of the Gaussian 09 program [52] using the Becke3LYP functionals [53,54,55] and double-zeta 6–31++G(d,p) basis sets [56]. The conformational structure of these drugs was also evaluated in solvent. The effect of solvent (water) was computed using the polarizable continuum method within the conductor-like polarizable continuum model (CPCM) [57,58,59]. The geometries of all drugs were fully optimized. For calculations of macroscopic p*K*a values, we used the program SPARC [60,61,62,63]. In the absence of the experimental single-crystal X-ray data about the molecular conformations of most of the antiplatelet drugs studied, the initial conformations to be used in the calculations were designed by using the Gauss View program and the available X-ray data for similar compounds [15,17,18].

## 4. Conclusions

(i) The density functional Becke3LYP method has been applied to study the molecular structure of ten species representing prodrugs and drugs acting at the P2Y12 platelet receptor. The fully-optimized most stable conformers of the thienopyridine drugs ticlopidine, clopidogrel and ticlopidine in both gas-phase and water solution are conformers with a mutual *gauche* orientation of phenyl and thienopyridine rings.
(ii) Of the indirect irreversible thienopyridine drugs, ticlopidine is the most basic molecule. The basicity decreases after its metabolization in the liver. Therapeutically-active metabolites of ticlopidine, clopidogrel and prasugrel are organic acids with p*K*as in the range of 3–3.4, and at pH = 7.4, they exist in the dissociated form only. The acidic sulfonamide group of the direct antiplatelet agent elinogrel is completely dissociated at physiological pH. Both ticagrelor and its active metabolite are present at pH = 7.4 in the neutral undissociated form. Cangrelor contains three acidic phosphate groups with p*K*as in the range of 0.33–0.59, which are completely dissociated at blood pH.
(iii) A trend in the drug lipophilicity was also observed. It is lowest for the intravenous agents cangrelor and elinogrel. The studied antiplatelet agents are only slightly soluble in water. Prodrug activation in thienopyridines results in the lowering of lipophilicity in comparison with parent prodrugs and in improving their solubility characteristics.
(iv) Polar surface area, owing to the heterogeneous character of these antiplatelet drugs, shows very large intervals of values (Table 5). Thienopyridine prodrugs, like ticlopidine, clopidogrel and prasugrel, with the lowest PSA values, exhibit the largest absorption.

## Figures and Tables

**Table 1:** Optimized dihedral angles (degrees) of the antiplatelet drugs studied. CPCM, conductor-like polarizable continuum model.

Dihedral Angle ^a^	X-Ray from PDB		X-Ray, Solid State Structure of Drug	B3LYP	B3LYP-CPCM
**Ticlopidine**	pdb.3KW4				
α[C(1)–C(2)–C(3)–N(4)]	79.2 ^b^		−98.07 ^c^	74.13	75.37
β[C(2)–C(3)–N(4)–C(5)]	179.5 ^b^		66.32 ^c^	69.62	69.01
**Ticlopidine metabolite**					
α[C(1)–C(2)–C(3)–N(4)]				178.43	
β[C(2)–C(3)–N(4)–C(5)]				−71.86	
γ[C(5)–C(6)–C(7)–C(8)]				175.34	
δ[C(6)–C(7)–C(8)–O(9)]				−8.10	
**Clopidogrel**	Pdb.4H1N	Pdb.3ME6			
α[C(1)–C(2)–C(3)–N(4)]	−122.0 ^d^	−104.6 ^e^	−117.4 ^f^	−127.92	−128.36
β[C(2)–C(3)–N(4)–C(5)]	87.5 ^d^	159.3 ^e^	60.7 ^f^	59.80	57.57
γ[C(1)–C(2)–C(3)–C(6)]	95.9 ^d^	115.4 ^e^	122.7 ^f^	110.59	109.94
δ[C(2)–C(3)–C(6)–O(7)]	−134.9 ^d^	77.3 ^e^	107.8 ^f^	87.29	87.82
**Clopidogrel metabolite**					
α[C(1)–C(2)–C(3)–N(4)]				−134.75	
β[C(2)–C(3)–N(4)–C(5)]				63.03	
γ[C(1)–C(2)–C(3)–C(6)]				101.24	
δ[C(2)–C(3)–C(6)–O(7)]				−103.28	
ε[C(5)–C(8)–C(9)–C(10)]				175.05	
ζC(8)–C(9)–C(10)]–O(11)]				−8.37	
**Prasugrel**					
α[C(1)–C(2)–C(3)–N(4)]			−128.70 ^g^	−129.99	−124.68
β[C(2)–C(3)–N(4)–C(5)]			59.54 ^g^	58.59	54.90
γ[C(1)–C(2)–C(3)–C(6)]			109.27 ^g^	106.99	112.12
δ[C(2)–C(3)–C(6)–O(7)]			104.03 ^g^	98.87	98.59
ε[C(8)–C(9)–O(10)–C(11)]			165.16 ^g^	179.96	179.50
ζ[C(9)–O(10)–C(11)–O(12)]			2.96 ^g^	−0.35	0.19
R(C=O…S)			2.7242 ^g^	2.7835	2.7870
**Prasugrel metabolite**					
α[C(1)–C(2)–C(3)–N(4)]				−129.58	−125.23
β[C(2)–C(3)–N(4)–C(5)]				59.65	56.59
γ[C(1)–C(2)–C(3)–C(6)]				107.48	111.74
δ[C(2)–C(3)–C(6)–O(7)]				99.23	98.79
ε[C(5)–C(8)–C(9)–C(10)]				175.23	176.20
ζC(8)–C(9)–C(10)]–O(11)]				−8.25	−7.71
**Elinogrel**					
α[C(1)–N(2)–C(3)–C(4)]				87.30	89.53
β[C(5)–C(6)–N(7)–C(8)]				−1.14	−2.10
γ[C(6)–N(7)–C(8)–N(9)]				177.18	178.51
δ[N(7)–C(8)–N(9)–S(10)]				−35.94	−29.38
ε[C(8)–N(9)–S(10)–C(11)]				−65.63	−71.85
ζ[N(9)–S(10)–C(11)–S(12)]				−85.49	−81.97
N–H…O=S				2.016	2.001
S=O…S				3.185	3.221
**Ticagrelor**					
α[N(1)–C(2)–N(3)–C(4)]				5.18	2.96
β[C(2)–N(3)–C(4)–C(5)]				−151.08	−152.62
γ[N(6)–N(7)–C(8)–C(9)]				124.16	122.87
**Ticagrelor metabolite**					
α[N(1)–C(2)–N(3)–C(4)]				6.18	4.51
β[C(2)–N(3)–C(4)–C(5)]				−153.39	−154.98
γ[N(6)–N(7)–C(8)–C(9)]				127.01	121.08
**Cangrelor**					
α[N(1)–C(2)–N(3)–C(4)]				−179.47	176.59
β[C(5)–N(6)–C(7)–O(8)]				−52.23	−44.70
γ[N(6)–C(7)–O(8)–C(9)]				−167.89	−163.29
δ[C(7)–O(8)–C(9)–C(10)]				150.31	142.76
ε[O(8)–C(9)–C(10)–O(11)]				56.23	52.38
ζ[C(9)–C(10)–O(11)–P(12)]				56.13	59.20
η[C(10)–O(11)–P(12)–O(13)]				−75.81	−84.16
ν[O(11)–P(12)–O(13)–P(14)]				−118.54	−119.70
θ[P(12)–O(13)–P(14)–C(15)]				−88.98	−88.60
μ[O(13)–P(14)–C(15)–P(16)]				47.72	48.71
ξ[P(14)–C(15)–P(16)–O(17)]				37.35	36.18
Cangrelor Tetrasodium					
α[N(1)–C(2)–N(3)–C(4)]				175.92	175.10
β[C(5)–N(6)–C(7)–O(8)]				−18.98	−13.45
γ[N(6)–C(7)–O(8)–C(9)]				−168.76	−164.60
δ[C(7)–O(8)–C(9)–C(10)]				168.33	164.39
ε[O(8)–C(9)–C(10)–O(11)]				65.33	66.26
ζ[C(9)–C(10)–O(11)–P(12)]				92.66	108.14
η[C(10)–O(11)–P(12)–O(13)]				−80.04	−69.87
ν[O(11)–P(12)–O(13)–P(14)]				−54.85	−76.47
θ[P(12)–O(13)–P(14)–C(15)]				−154.46	−135.71
μ[O(13)–P(14)–C(15)–P(16)]				60.37	63.52
ξ[P(14)–C(15)–P(16)–O(17)]				160.82	169.00

^a^ For the definition of dihedral angles, see Figure 1; ^b^ [14]; ^c^ [15]; ^d^ [16]; ^e^ [14]; ^f^ [17]; ^g^ [18].

**Table 2:** The solvation energies (water) and dipole moments of the drugs studied.

Drug	ΔE^CPCM^, kJ/mol	μ, Debye (D)
Ticlopidine	−20.4	2.19
Ticlopidine active metabolite	−35.2	3.46
*(S)-*Clopidogrel	−29.2	1.75
*(S)-*Clopidogrel active metabolite	−48.8	3.88
*(S)-*Prasugrel	−39.6	3.94
*(S)-*Prasugrel active metabolite	−46.2	2.86
Elinogrel	−96.9	11.01
Ticagrelor	−76.75	5.36
Ticagrelor active metabolite	−73.7	4.32
Cangrelor	−91.9	2.23
Cangrelor tetrasodium	−1316.2	14.44

**Table 3:** The relative stability and dipole moments of the four stereoisomers of the prasugrel metabolite R-138727.

Position ^1^	Stereoisomer	ΔE, kJ/mol	ΔE^CPCM^, kJ/mol	μ ^2^, Debye (D)
a,b	*S,S*	0	0	3.55
a,b	*R,S*	41.8	32.5	4.56
a,b	*S,R*	18.9	17.4	3.83
a,b	*R,R*	51.8	40.8	3.19

^1^ For the position of asymmetric carbons, see Figure 1; ^2^ gas-phase dipole moment.

**Table 4:** Calculated p*K*a values (at 25° C) of the antiplatelet drugs investigated.

Drug	p*K*a		% Ionized Form	
	Acid Function	Basic Function	Acid Function	Basic Function
Ticlopidine		7.85		74
Ticlopidine active metabolite	3.41; 9.43	7.26	100; 1	42
Clopidogrel		4.61		0.2
Clopidogrel active metabolite	3.01; 9.15	4.43	100; 2	0.1
Prasugrel		5.50		1
Prasugrel active metabolite	3.29; 9.19	5.06	100; 1.5	0.5
Elinogrel	4.26; 11.37		100; 0	
Ticagrelor	13.48	2.28	0	0
Ticagrelor active metabolite	13.27	2.59	0	0
Cangrelor	0.33–0.59		100	

**Table 5:** Calculated lipophilicity (clog*P*), solubility (clog*S*), absorption (%ABS), polar surface area (PSA) and Lipinski parameters of the antiplatelet drugs studied ^a^.

Drug	Log*P* ^b^, Exp	Clog*P* (XLOGP2)	log*D* (pH = 7.4)	ClogS (AClogS)	%ABS	logS Exp	Volume Å^3^	PSA Å^2^	NROTB	*n* ON Accept- ors	*n* OHNH Donors	Formula Weight (Da)
Ticlopidine	2.9	2.93		−3.55 (74.70 mg/L)	107.9		228.85	3.24	2	1	0	263.78
Ticlopidine active metabolite		2.39	−0.87	−3.05 (0.27 g/L)	95.0		255.64	40.53	3	3	1	297.81
Clopidogrel	2.5	2.50		−3.22 (0.19 g/L)	98.8		273.40	29.54	4	3	0	321.82
Clopidogrel active metabolite		1.96	−1.20	−2.72 (0.68 g/L)	85.9		300.19	66.84	4	5	1	355.84
Prasugrel	3.53	1.97		−4.08 (30.80 mg/L)	92.9		323.37	46.61	6	4	0	373.44
Prasugrel active metabolite		1.65	−1.07	−3.13 (0.26 g/L)	89.1		305.63	57.61	5	4	1	349.43
Elinogrel		1.89	−1.80	−6.55 (0.15 mg/L)	59.9		392.53	142.16	5	10	4	523.95 (viol.)
Ticagrelor		1.95		−5.21 (3.24 mg/L)	61.2	−4.71 (10 mg/L)	438.27	138.45	10	10	4	522.56 (viol.)
Ticagrelor active metabolite		2.36		−5.29 (2.47 mg/L)	64.4		395.68	129.21	7	4	9 (viol.)	458.52
Cangrelor		−0.41	−5.86	−3.89 (99.10 mg/L)	20.7		541.61	255.92	16	17 (viol.)	7 (viol.)	776.37 (viol.)

^a^ for definition of molecular descriptors see [46,47]; ^b^ www.drugbank.ca.

**Table 6:** Drug-like properties of the antiplatelets investigated.

Molecular Descriptor	Prodrug	Drug (Active Metabolite)
Molecular weight (*M*w)	260–530	300–450
Octanol/water partition coefficient (clog*P*)	1.9–2.9	1.7–2.4
Aqueous solubility (clog*S*)	−6.5–(−3.2)	−5.3–(−2.7)
Polar surface area (PSA, Å^2^)	3–140	40–130
Percent of oral absorption (%ABS)	60–107	65–95

## Supplementary Materials

Supplementary materials can be found at http://www.mdpi.com/1422-0067/17/3/388/s1.
