# Supplementary material for: A Comparative Study of Molecular Structure, pKa, Lipophilicity, Solubility, Absorption and Polar Surface Area of Some Antiplatelet Drugs
Source: Int J Mol Sci. 2016 Mar 19;17(3):388. doi: 10.3390/ijms17030388 (PMC4813244; doi:10.3390/ijms17030388)
Supplement: Supplementary file 1 [file ijms-17-00388-s001.pdf]

Supplementary Materials: A Comparative Study of Molecular Structure, p*K*a, Lipophilicity, Solubility, Absorption and Polar Surface Area of Some Antiplatelet Drugs

Milan Remko, Anna Remková and Ria Broer

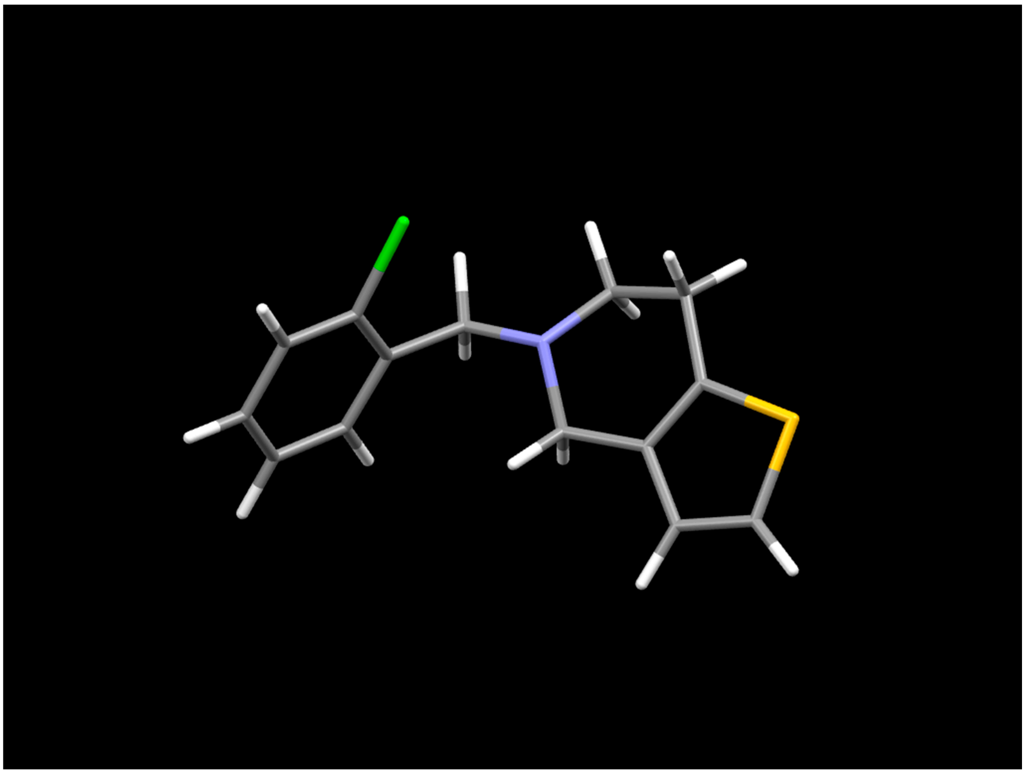

Overall shape of the ticlopidine computed at the Becke3LYP/6-311++G(d,p) level of theory.

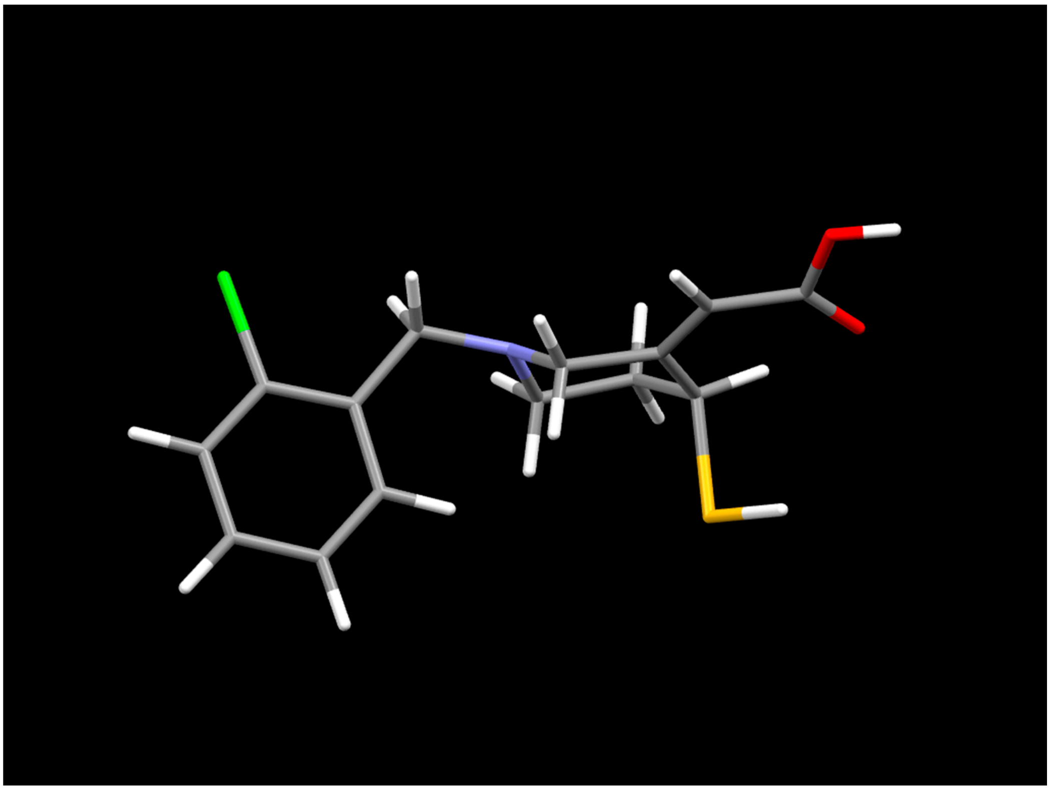

Overall shape of the ticlopidine metabolite computed at the Becke3LYP/6-311++G(d,p) level of theory.

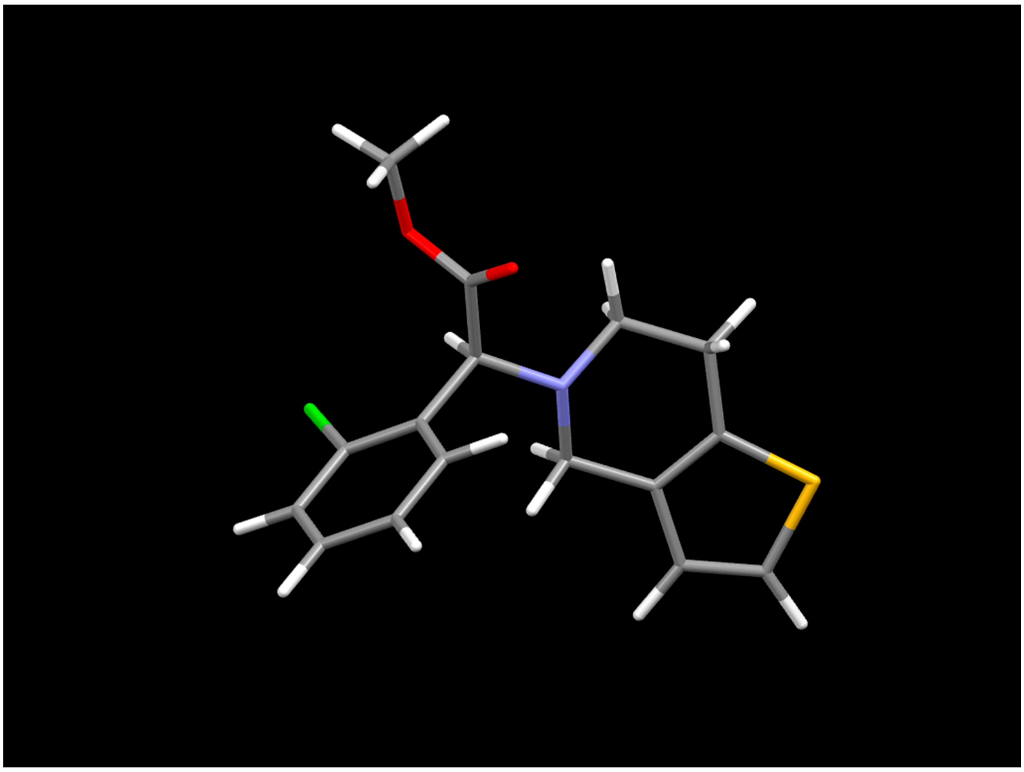

Overall shape of the clopidogrel computed at the Becke3LYP/6-311++G(d,p) level of theory.

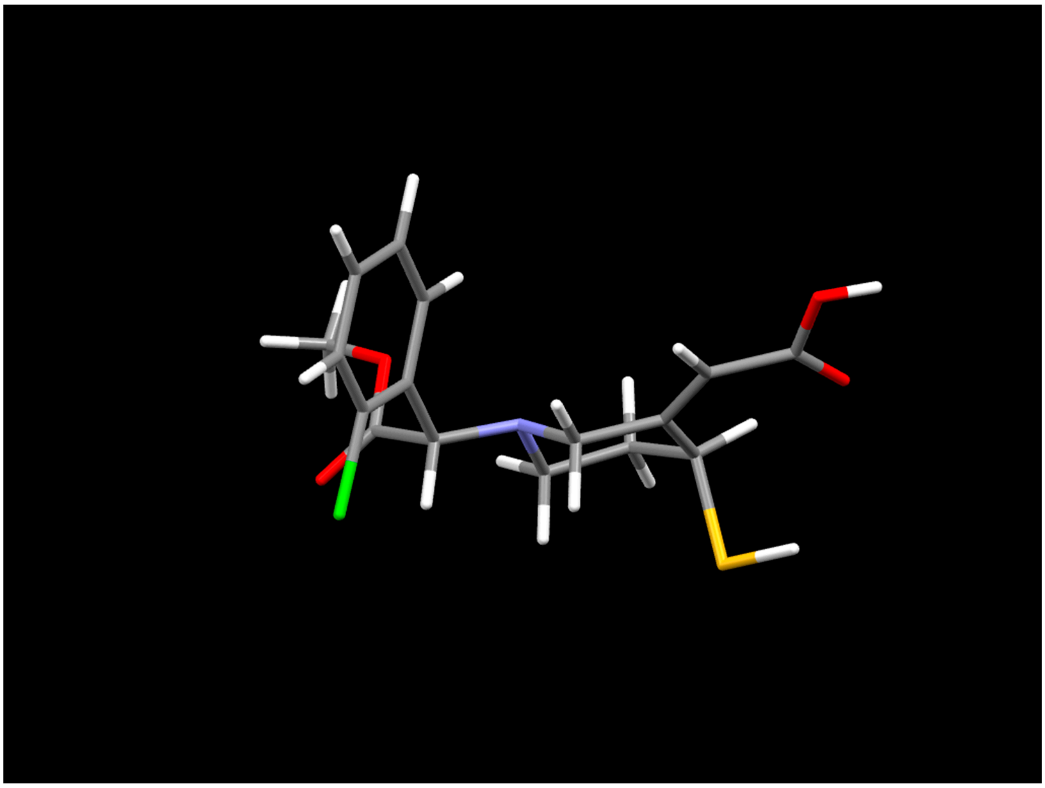

Overall shape of the clopidogrel metabolite computed at the Becke3LYP/6-311++G(d,p) level of theory.

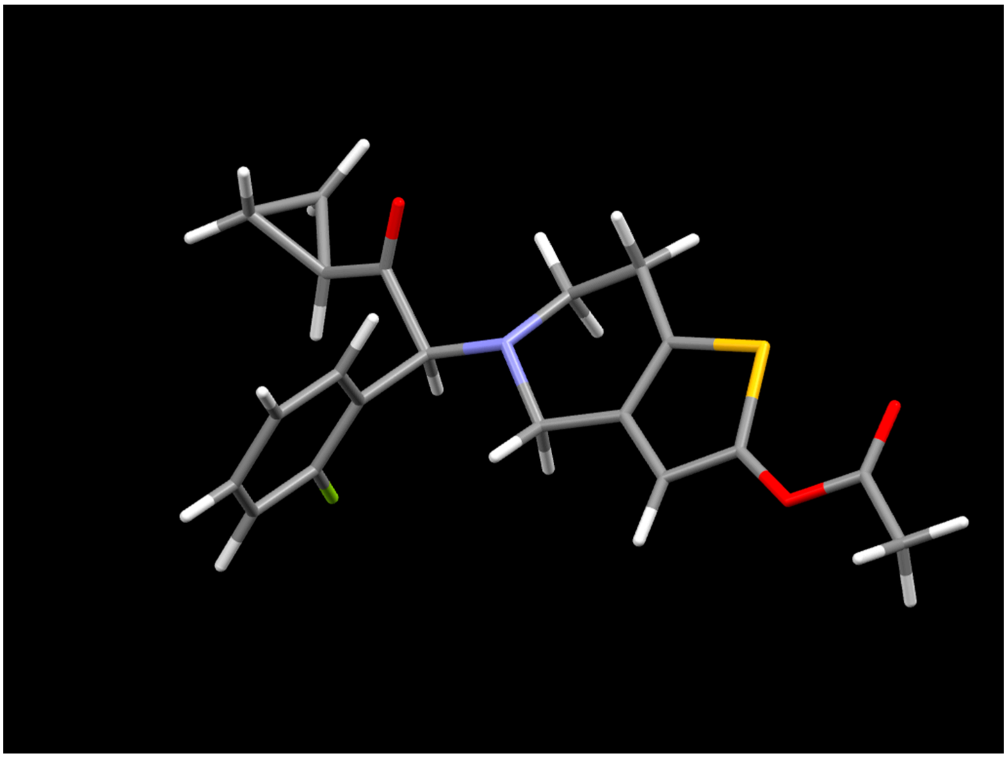

Overall shape of the prasugrel computed at the Becke3LYP/6-311++G(d,p) level of theory.

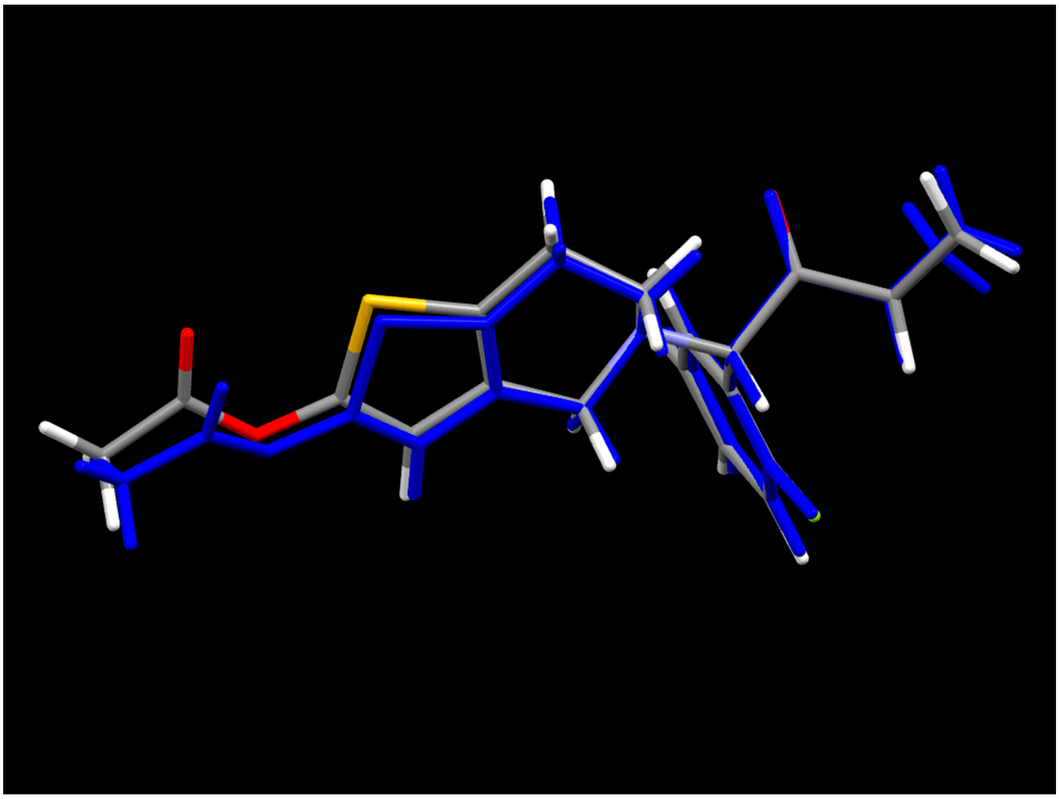

Molecular superimposition of the Becke3LYP-optimized molecular structure of prasugrel (color by connected atoms) and solid state prasugrel from [18] (blue).

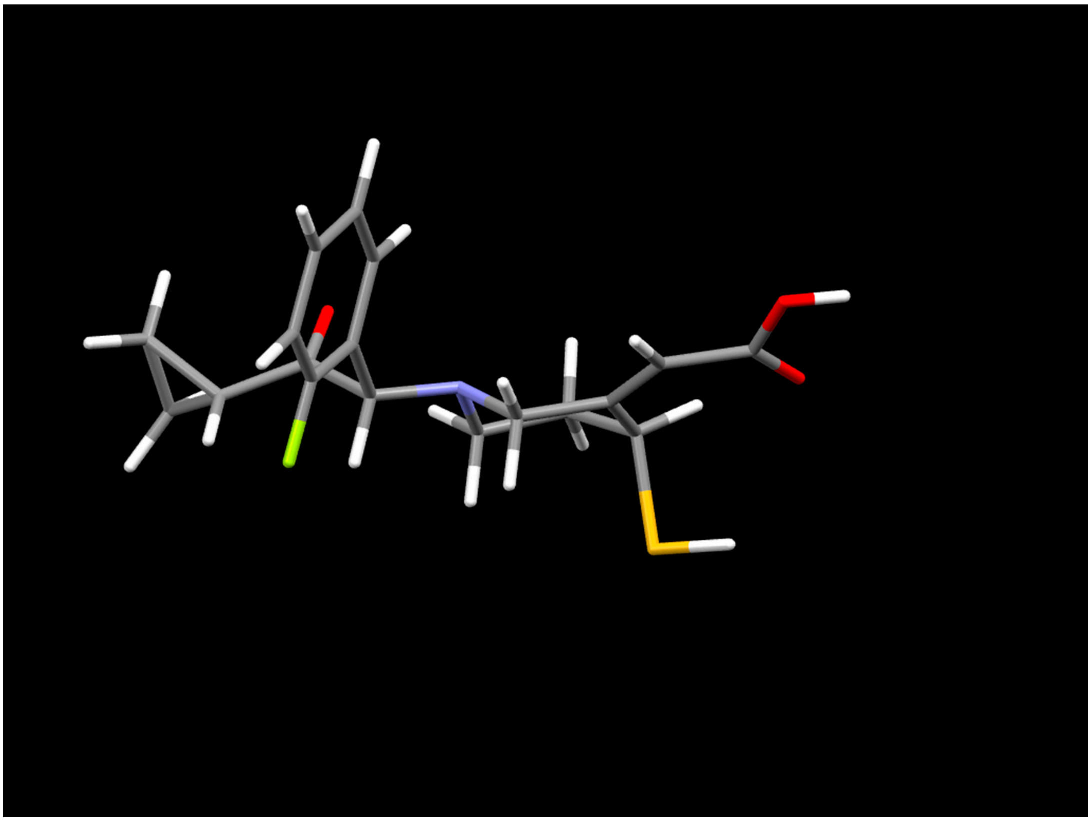

Overall shape of the prasugrel metabolite computed at the Becke3LYP/6-311++G(d,p) level of theory.

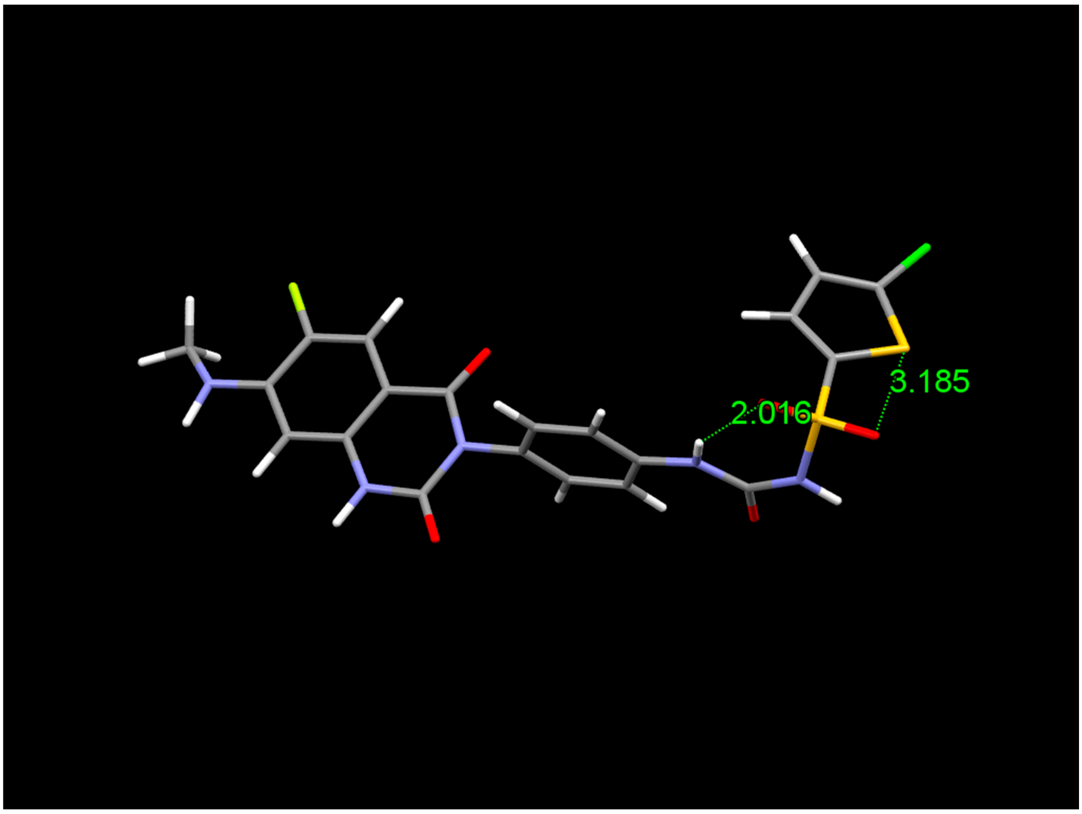

Overall shape of the elinogrel computed at the Becke3LYP/6-311++G(d,p) level of theory.

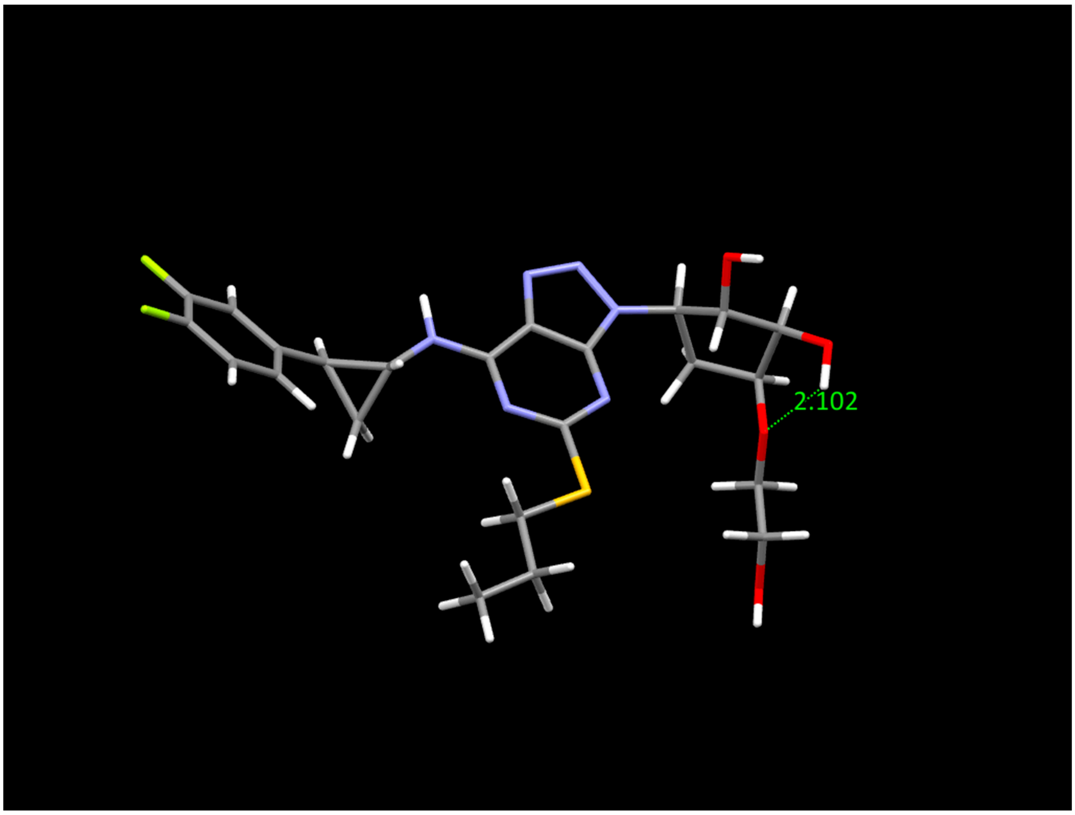

Overall shape of the ticagrelor computed at the Becke3LYP/6-311++G(d,p) level of theory (bond lengths are in Angstroms).

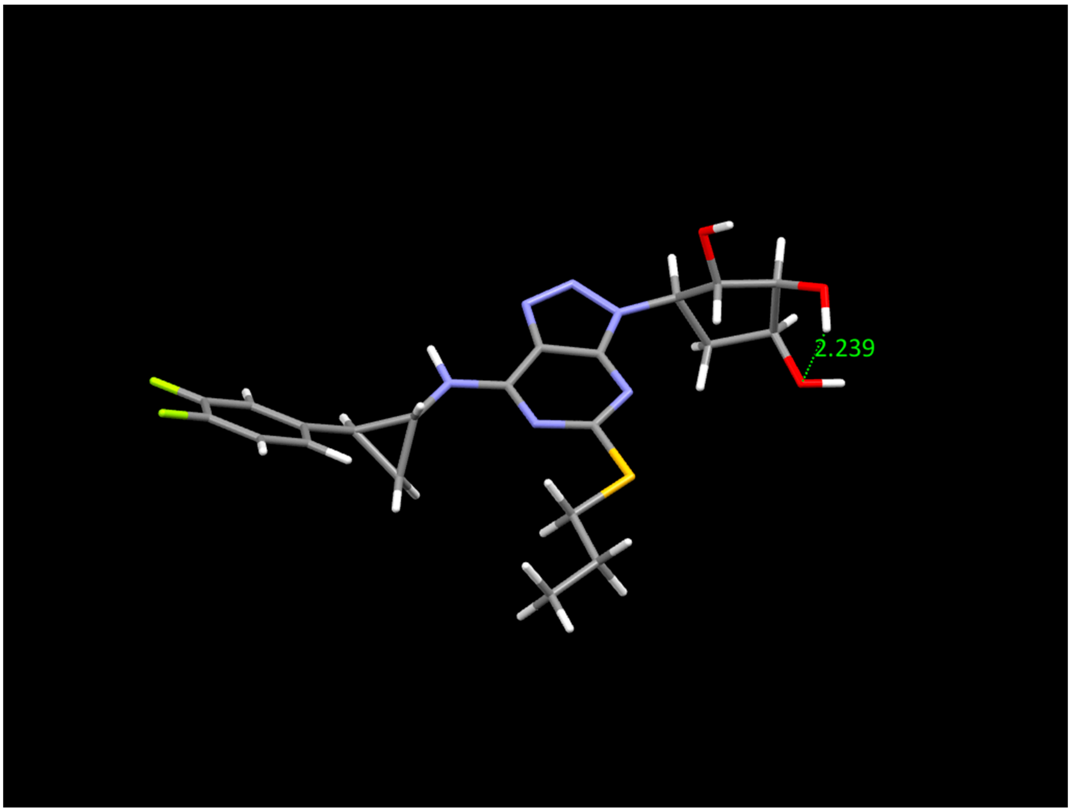

Overall shape of the ticagrelor metabolite computed at the Becke3LYP/6-311++G(d,p) level of theory (bond lengths are in Angstroms).

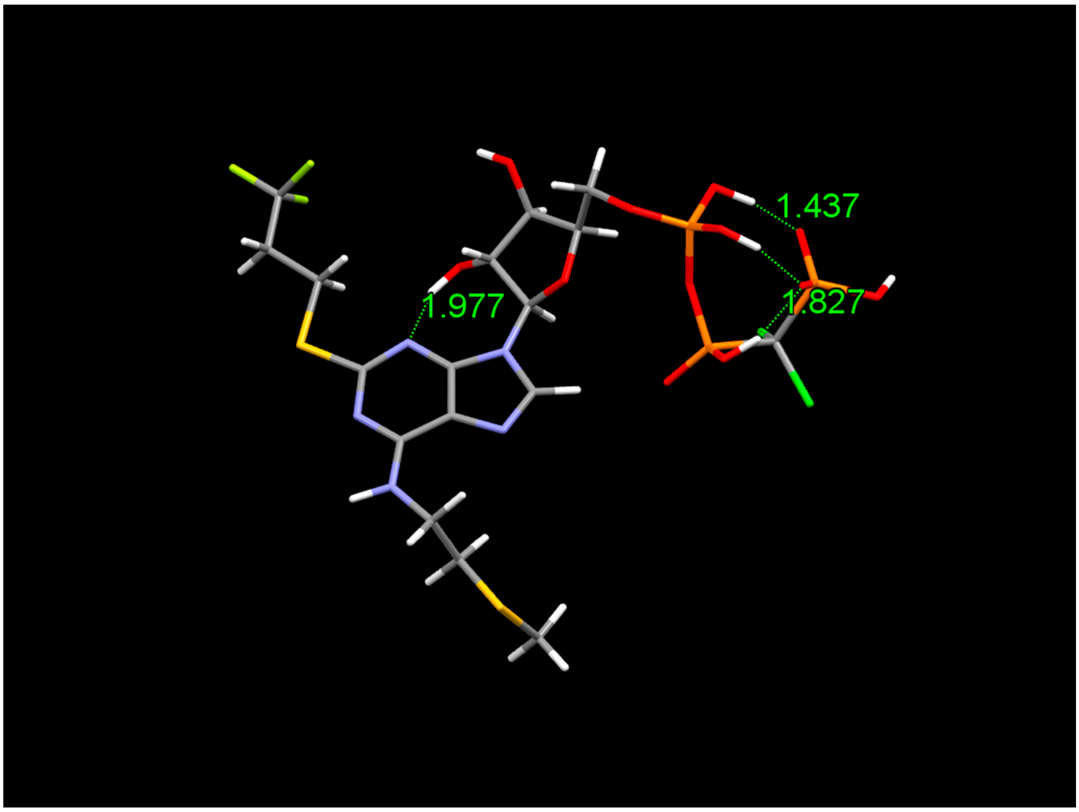

Overall shape of the cangrelor computed at the Becke3LYP/6-311++G(d,p) level of theory (bond lengths are in Angstroms).

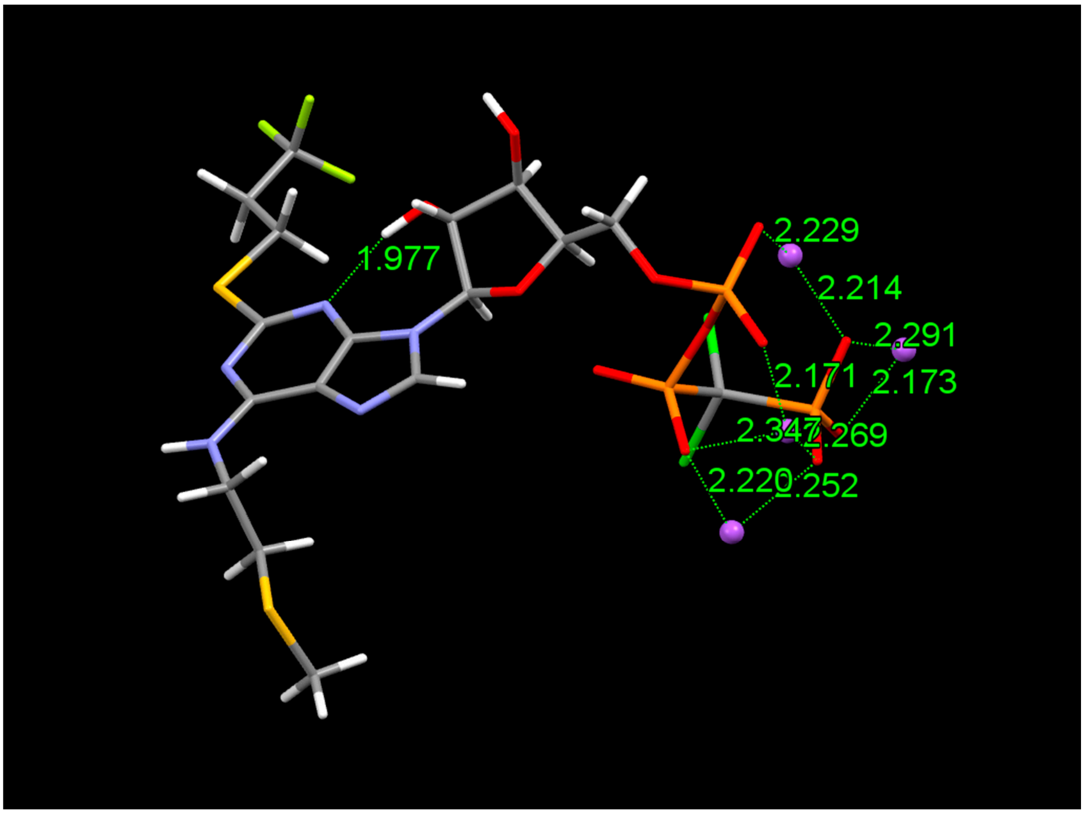

Overall shape of the cangrelor tetrasodium computed at the Becke3LYP/6-311++G(d,p) level of theory (bond lengths are in Angstroms).

Figure S1. The geometries of the drugs studied. Molecule figures were generated using Mercury software (http://www.ccdc.cam.ac.uk/mercury/).
